# Preparation, Air Filtration Performance of a Fluorinated Polyimide/Polyacrylonitrile Nanofibrous Membrane by Electrospinning

**DOI:** 10.3390/polym16091240

**Published:** 2024-04-29

**Authors:** Chen Chen, Lulu Xiong, Yahui Cui, Chaosheng Wang

**Affiliations:** 1 School of Materials Science and Engineering, Shanghai University of Engineering Science, Shanghai 201620, China; 2 Key Laboratory of High Performance Fibers & Products, Ministry of Education, Donghua University, Shanghai 201620, China; 3 Shanghai Dajue Packaging Products Co., Shanghai 201620, China; 4 Energy Bureau of Xiangyuan County, Changzhi 046200, China

**Keywords:** polyacrylonitrile, fluorinated polyimide, electrospinning, air filter membrane, hydrophobicity

## Abstract

This paper reports the successful fabrication of a new nanofibrous membrane, F-PI/PAN, through electrospinning of polyacrylonitrile (PAN) and fluorinated polyimide (F-PI). The nanofibrous membrane exhibits comprehensive properties for high-temperature filtration and robust PM_2.5_ (particulate matter with an aerodynamic equivalent diameter of 2.5 microns or less) removal. The introduction of F enhances the hydrophobicity of the PI. The relationship between the hydrophobic performance and the filtration performance of particles is investigated. The chemical group of the composite membrane was demonstrated using FITR, while the surface morphology was investigated using field emission scanning electron microscopy. The TGA results indicated good thermal stability at 300 °C. Various ratios of F-PI membranes were prepared to characterize the change in properties, with the optimal mass ratio of F-PI being 20 wt%. As the proportion of F-PI increases, its mechanical and filtration efficiency properties and hydrophobicity become stronger. The contact angle reaches its maximum of 128 ± 5.2° when PAN:F-PI = 6:4. Meanwhile, when PAN:F-PI = 8:2, the filtration efficiency reaches 99.4 ± 0.3%, and the elongation at break can reach 76%. The fracture strength can also reach 7.1 MPa, 1.63 times that of the pure PAN membrane.

## 1. Introduction

With the rapid development of industrialization and urbanization, particulate matter (PM) pollution in the air [1,2], especially the notorious PM_2.5_ (particulate matter with an aerodynamic diameter ≤ 2.5 µm), which was listed as the first category of carcinogens by the World Health Organization, causes a growing impact on visibility, climate, and human health and quality of life [3,4,5,6,7]. They come in many forms, such as dust, mist, fumes, smoke, and fog, which are produced not only by nature but also through human activities [8]. Therefore, it is essential to develop high-performance filters with high filtration efficiency, low energy costs, and a long service life to combat severe air pollution.

Currently, the most commonly used air filter materials on the market are nonwoven fabrics, glass fibers, and melt-blown electret fibers. Fibrous filters are the most cost-effective and universal [9,10,11,12]. Electrospinning technology has been widely used in this field due to the small fiber diameters and high porosity of its membrane. Polyacrylonitrile (PAN) is a commonly used raw material in electrospinning. The spinning conditions of PAN are relatively mild [13,14,15]. Moreover, there are cyanogen polar functional groups in PAN molecules that show a binding solid force with particulate matter [16]. Therefore, it can be used as the primary material for filter media [17]. On the other hand, basic polyimide (PI) materials for high-temperature filtration have received increasing attention. For instance, Shang Leiming et al. [18] successfully prepared a high-temperature resistant composite nanofiber filter mat with an aramid nonwoven mat as the substrate and polyimide nanofibers with a diameter of 150–400 nm as the high-temperature filter layer. This fiber mat can filter NaCl particles with a diameter between 1.0 and 2.0 microns and greater than 2.0 microns with a filtration efficiency of 99.5% and 100%, respectively. Gao et al. [19] compared filters containing 15 wt% PI nanofibers with three commercial filter materials and found that the pressure drop of the commercial air filters was much higher than that of PI air filters. Although Com-1# and Com-2# commercial air filters have high PM removal efficiency, their pressure drop is too high to achieve high airflow. In a real environment, PI air filters were applied to automobile exhaust particulate filters and remained highly effective for particle removal even after 120 h of continuous work [20]. In numerous studies, polyimides have shown excellent air filtration performance and high-temperature stability [21,22,23,24,25,26].

However, the fibrous filter material’s performance and filtration efficiency still have limitations. It is well known that increasing the material’s hydrophobicity only partially improves the fiber’s ability to filter particulate matter [27,28,29,30]. Fluorine atoms are known to be the strongest in the periodic table regarding electronegativity [31,32,33]. Introducing fluorine atoms tends to cause lipophilic materials to exhibit excellent hydrophobic properties [34]. Park et al. [35] introduced fluorine atoms into nanofiber membranes. The fiber membrane has excellent hydrophobic performance and long-term stable operation over 250 h. Myeong et al. [36] prepared a hydrophobic mesh filter with excellent separation efficiency and separation speed. Hydrophobicity is improved by endowing surface fluorine functional groups with fluorine plasma. The relationship between the material’s hydrophobic performance and its ability to filter particles has yet to be extensively researched.

In this paper, we discuss the relationship between the hydrophobic performance and the filtration performance of particles to improve the filtration performance of nanofibrous membranes. In combination with the excellent comprehensive performance of polyimide, an F-PI precursor solution was prepared by introducing trifluoromethyl (−CF_3_) into polyimide. The final solution was then made by blending F-PI and PAN. Electrospinning technology was used to prepare F-PI/PAN composite nanofibers. The results indicate that as the proportion of F-PI increases, the mechanical properties, filtration efficiency, and hydrophobicity also increase.

## 2. Materials and Methods

### 2.1. Materials

TFMB (2,2′-bis(trifluoromethyl)-4,4′-diaminobiphenyl) and 6FDA (4,4′-(hexafluoroisopropylidene)diphthalic anhydride) were purchased from Changzhou Sunshine Pharmaceutical Co., Ltd., Changzhou, China. PAN (Polyacrylonitrile, Mn = 20 × 10^4^), NMP (*N*-methyl-2-pyrrolidone) and DMF (*N*,*N*-dimethyl formamide) were provided by Sinopharm Group Chemical Reagent Co., Ltd., Shanghai, China. All chemicals were of analytical grade and were used as received without further purification.

### 2.2. Preparation of Fluorinated PI/PAN Nanofibrous Membrane

#### 2.2.1. Preparation of Fluorine-Containing Polyimide Acid Solution

**Scheme 1:**
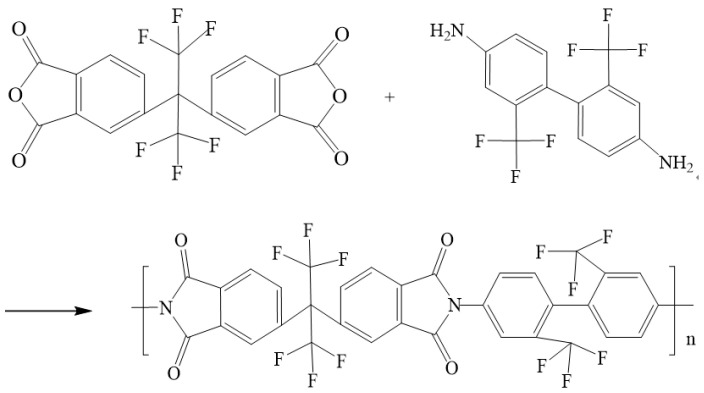
Synthesis process of fluorine-containing polyimide (F-PI).

The molar ratio of TFMB and 6FDA was 1:1. The spinning solution with 20 wt% was prepared in NMP solvent. After TFMB was dissolved completely in the solvent, 6FDA was added, and the polyimide acid spinning solution was synthesized under nitrogen protection at 3 °C. The resulting reaction solution was stored in the refrigerator for further reaction. The reaction mechanism is shown in Scheme 1.

#### 2.2.2. Preparation of PAN Solution

To obtain a polyacrylonitrile solution with a solid content of 14 wt%, DMF and polyacrylonitrile were added to a three-port flask. The overhead mixer was then adjusted to the appropriate rotating speed and stirred for 4–6 h under nitrogen gas protection until a colorless and transparent solution was obtained.

#### 2.2.3. Preparation of F-PAA/PAN Solution

The PAA/PAN mixed solution was prepared using the blending method. The PAA/PAN mixed solution was prepared using the physical blending method. The polyacrylonitrile and F-polyimide acid solutions were mixed in mass ratios of 10:0, 9:1, 8:2, 7:3, and 6:4, respectively, while being mechanically stirred for approximately 4–6 h under nitrogen protective gas. The resulting solution was fully and evenly mixed. The PAA/PAN mixed solution was prepared using the physical blending method.

#### 2.2.4. Preparation of F-PAA/PAN Nanofibrous Membrane

An adequate amount of spinning solution was used for electrospinning. The spinning process parameters were as follows: the advance speed of the spinning machine was 0.0010 mm/h, the applied voltage was 15 kV, the receiver roller speed was 50 r/min, the ambient temperature was 35 ± 5 °C, and the humidity was 40 ± 5%RH.

#### 2.2.5. Preparation of Fluorinated PI/PAN Nanofibrous Membrane

The fluorinated PI/PAN nanofibrous membrane was vacuum-dried for 6 h before undergoing high-temperature imidization treatment in a vacuum tube furnace. The imidization process was as follows: the heating rate was set at 5 °C/min, and the temperature was kept at 110 °C, 150 °C, 200 °C, and 250 °C for 1 h each. Finally, the temperature was raised to 300 °C for 0.5 h, and then the iminization process was completed by allowing it to cool naturally to room temperature. This produced a complete polyimide nanofibrous membrane, which was then tested for performance.

### 2.3. PM Generation and Evaluation of the Filtering Efficiency

**Scheme 2:**
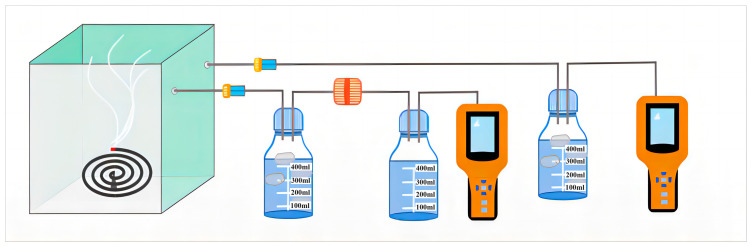
Laboratory homemade air filtration simulator.

A self-made device shown in Scheme 1 was used to measure the filtering efficiency of the nanofibrous membrane. In this paper, the smoke of burning incense was used to simulate external air pollution because it includes CO, CO_2_, NO_2_, and SO_2_, as well as volatile organic compounds such as benzene, toluene, xylene, aldehydes, and polycyclic aromatic hydrocarbons, in addition to small particles of various sizes, which are similar to air pollutants in the air. As shown in Scheme 2, the incense was in a closed box connected to two gas detectors (Intelligent Gas Detector PGM-300) fitted with a peristaltic pump. When fitted with air filters of appropriate size, these detectors measure total particulate matter and the PM_1_, PM_2.5_, PM_5_, and PM_10_ concentrations. The diameter of the air filters employed in this study was 12 cm. One gas detector (GD-2) directly connected to the bottle serves as a control, measuring total PM_2.5_ concentration in the closed bottle without filtration, and the other gas detector (GD-1) connected through the membrane module measures PM_2.5_ concentration after filtration.

The efficiency of filtration was thus calculated. The data measured by GD-1 were recorded as n_1_, and the data measured by GD-2 were recorded as n_2_. In other words, the concentration of PM_2.5_ before filtration was n_1_ mg/m^3^, and the concentration of PM_2.5_ after fiber membrane filtration was n_2_ mg/m^3^. The calculation formula for filtration efficiency is as follows:

η = (1 − n_1_/n_2_)×100% (1)

**Scheme 3:**
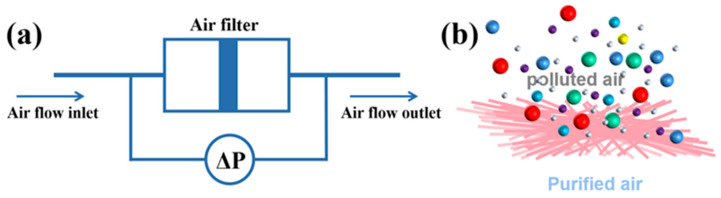
Mechanism diagram of the pressure drop test (**a**) and the air filtration model (**b**).

Then, to monitor the pressure drop during filtration, a differential pressure transmitter (Digital Manometer SD20, Fast electronic technology Co., Ltd., Guangzhou, China) was connected across the membrane module, as shown in Scheme 3. Meanwhile, the quality factor (QF) of the air filters employed in this study was calculated as follows:

(2) $$QF=-\frac{\ln\left(1-\eta \right)}{\Delta p}$$

where η is the PM_2.5_ filtration efficiency and ΔP is the air drop value across the air filters.

### 2.4. Characterization

The micrographs of the membrane were obtained by field-emission scanning electron microscopy (S-8010, Hitachi, Tokyo, Japan). Before the observation, all the samples were coated with gold. The average diameter distribution was plotted using the software Nano Measure (Version 1.2). The chemical structure of the membrane was analyzed by Fourier transform infrared (FTIR) spectroscopy using a Nicolet iS50 instrument (Thermo Fisher Scientific, Waltham, MA, USA), with the spectral range of the test usually being 4000–500 cm^−1^; the resolution of the instrument is 16 cm^−1^. The number of scans was 32. The samples were calcined in a single-temperature-zone open vacuum tube furnace (OTF-1200X) by Hefei Kejing Materials Technology Co., Ltd., Hefei, China. The thermal stability of the membrane was tested using a SPA PT1000 thermogravimetric analyzer (Linseis, Zerb, Germany). The contact angle test was accomplished using an optical contact angle measuring instrument at room temperature with a DSA30 instrument (Kruss GmbH, Hamburg, Germany). The average value is taken as the result after obtaining the parameters of several measurement samples. A self-made air filtration device tested the filtration performance of the composite nanofibrous membrane. The tensile properties of the membrane were tested by a universal strength tester (Instron 6025, Jinan Liangong Testing Technology Co., Ltd., Jinan, China). The sample was cut into an 8 × 60 mm piece with a thickness of 30 μm, the clamping distance was 20 mm, and the stretching speed was 5 mm/min. Each sample was subjected to five parallel tests, and the values were averaged.

## 3. Results

### 3.1. Structural Characterization of Fluorinated PI/PAN Nanofibrous Membranes

**Figure 1:**
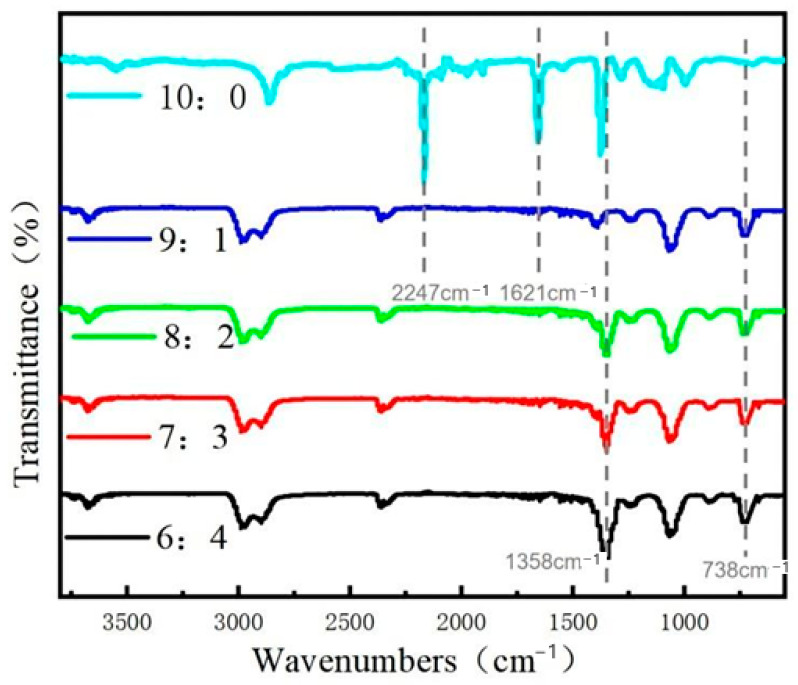
FTIR spectra of nanofiber membranes prepared with different PAN:F-PI ratios.

The FTIR of the nanofibrous membrane is shown in Figure 1. The peaks at 1358 cm^−1^, and 738 cm^−1^ are typical characteristic peaks of polyimide, of which 1358 cm^−1^ is the stretching vibration of the imide ring C−N, and 738 cm^−1^ is the deformation vibration of the imide ring. Moreover, with the increase in PI content from 10% to 40%, the intensity of these two peaks gradually increases. 2247 cm^−1^ is the characteristic absorption peak of cyanide (−C≡N) in polyacrylonitrile, and the addition of polyimide weakens the peak and shifts to the left. The results indicate that the calcined polyamide acid/polyacrylonitrile nanofibrous membrane is eliminated into the PI/PAN composite nanofibrous membrane, and the structure of PAN is slightly changed with the addition of fluorinated PI (F-PI).

**Figure 2:**
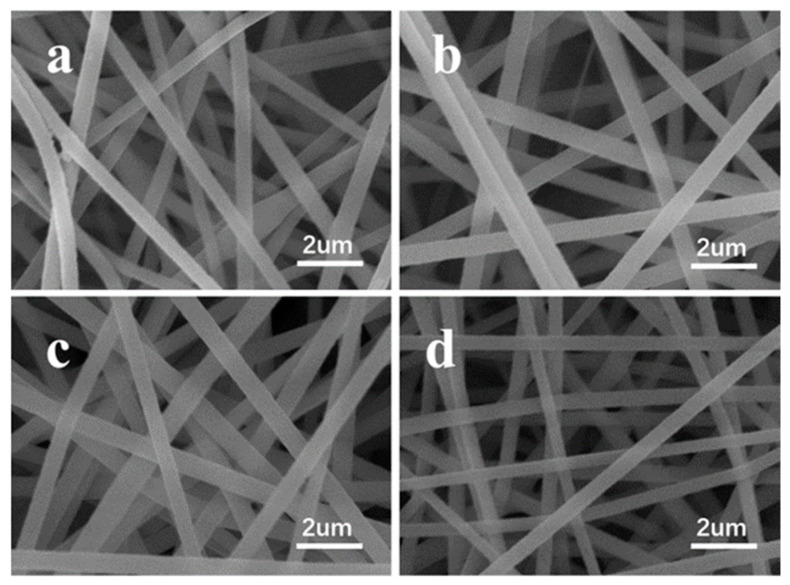
SEM image of nanofibrous membranes prepared with different ratios ((**a**) PAN:F-PI = 9:1, (**b**) PAN:F-PI = 8:2, (**c**) PAN:F-PI = 7:3, (**d**) PAN:F-PI= 6:4).

**Figure 3:**
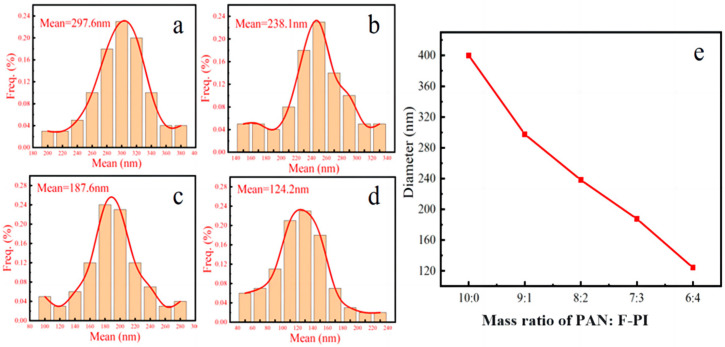
Distribution of fiber diameter in nanofibrous membranes prepared with different ratios ((**a**) PAN:F-PI = 9:1, (**b**) PAN:F-PI = 8:2, (**c**) PAN:F-PI = 7:3, (**d**) PAN:F-PI = 6:4) and diameter variation of nanofiber membranes prepared with different ratios (**e**).

Figure 2 shows the results of the scanning electron microscopy observation of the microscopic morphology of nanofibrous membranes. The fiber diameter distribution at four ratios is uniform, and no droplet or spray state exists. The nanomeasure software (Version 1.2) selected the F-PI/PAN pre-oxidized fiber film calcined in a tubular furnace to obtain the average diameter distribution, as shown in Figure 3. The average diameter of the nanofibrous membrane shows a decreasing trend. At a mass ratio of 9:1, the composite fiber has an average diameter of 297.6 nm. At a mass ratio of 8:2, the composite fiber has an average diameter of 238.1 nm. As the F-PI mass ratio increases, the average diameter of the fiber decreases, reaching a minimum of 187.6 nm at a constant increase. At an additional amount of 6:4, the fiber has a minimum average diameter of 124.2 nm. In conclusion, as the mass fraction of F-PI components in the solution increases, the diameter of the nanofibrous membrane decreases continuously. This can be attributed to the poorer spinning performance of the fluoride polyamide acid solution prepared in the laboratory, which causes droplets and sprays during spinning. Therefore, the introduction of F-PI not only improved the performance but also affected the diameter of the nanofibrous membrane.

### 3.2. Thermal Stability of Fluorinated PI/PAN Nanofibrous Membranes

**Figure 4:**
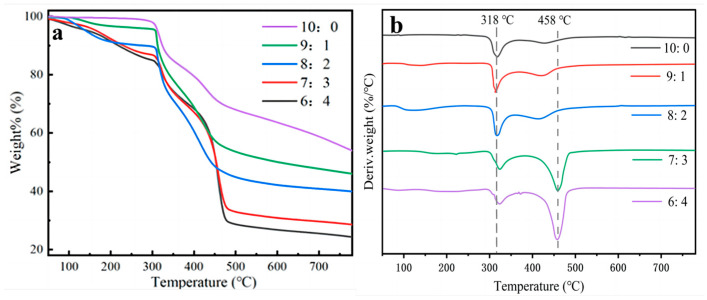
Thermal stability analysis of nanofibrous membranes prepared with different PAN:F-PI ratios ((**a**): TGA, (**b**): DTG).

Figure 4 shows the thermal stability of the nanofibrous membrane. Before 300 °C, the thermal weight loss rate of the nanofibrous membranes is slow due to the evaporation of solvents and the completion of imide. After 300 °C, the nanofibrous membrane begins to decompose chemically. The DTG chart shows a rapid decomposition process between 298 °C and 342 °C, mainly caused by breaking the PAN macromolecular chain. A second rapid decomposition occurs at around 450 °C, with a decomposition rate and temperature similar to that of pure PAN at a small amount of F-PI (9:1). As the content of F-PI increases, the decomposition rate gradually slows down. The chemical structure stability of polyimide is mainly due to its nitrogen-containing pentameric heterocyclic ring and aromatic ring. The second decomposition is caused by polyacrylonitrile dehydrocarbonization and polyimide macromolecules’ fracture decomposition.

### 3.3. Hydrophobic Performance of Fluorinated PI/PAN Nanofibrous Membranes

**Figure 5:**
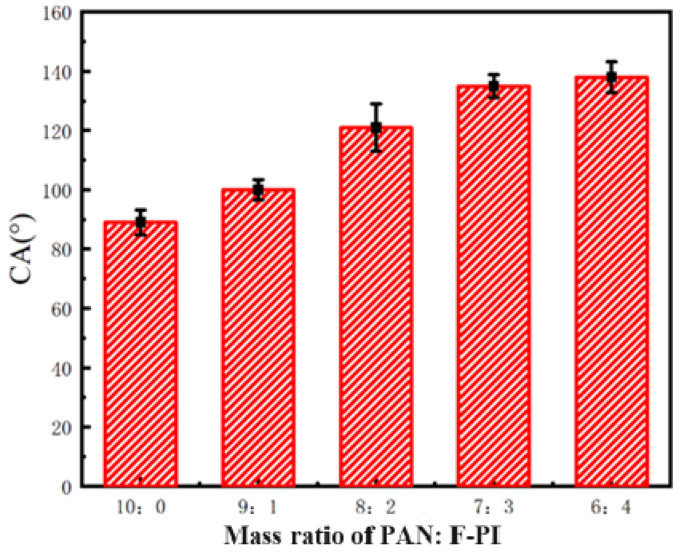
Contact angle of nanofibrous membranes prepared with different PAN:F-PI ratios.

Figure 5 shows the contact angle test results for the F-PI/PAN nanofibrous membrane. The hydrophobic performance of F-PI/PAN is better than that of PAN alone. The contact angle of the nanofibrous membrane is 101.3 ± 3.4°, 115.6 ± 7.9°, 125 ± 3.8°, and 128 ± 5.2° for mass fraction ratios of 9:1, 8:2, 7:3, and 6:4, respectively. The maximum contact angle was 128 ± 5.2° at 6:4. The addition of F-PI improved the hydrophobic properties of PAN itself.

### 3.4. Filtration Performance of Fluorinated PI/PAN Nanofibrous Membranes

**Figure 6:**
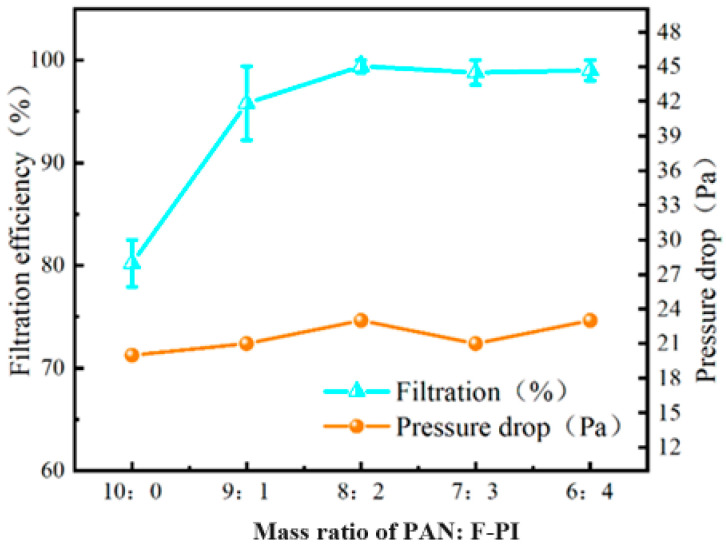
Filtration performance of nanofibrous membranes prepared with different PAN:F-PI ratios.

Filtration experiments were conducted using a self-made device and a nanofibrous membrane with a 1.0 g/m^2^ base weight. The results are presented in Figure 6. The pure PAN nanofibrous membrane had a filtration efficiency of only 80.2 ± 2.3% for PM_2.5_. However, with the addition of F-PI in a ratio of 9:1, the efficiency significantly increased to 95.8 ± 3.6%. Furthermore, the nanofibrous membrane’s filtration efficiency reached 99.4 ± 0.3% when the mass ratio of F-PI was 8:2. The efficiency of the blending process significantly improved due to the strong electronegativity of the fluorine atoms. The introduction of F increased the polarity of the polyimide, resulting in an uneven distribution of intramolecular charge and a greater dipole moment, which strengthened the molecular inter-atomic forces. Thus, the interaction between the composite nanofibrous membrane and particulate matter is enhanced, resulting in a significant improvement in the filtration performance of the composite nanofibrous membrane. The fluorocarbon bond, which has a very low surface energy due to the fluorine atom, currently provides the strongest hydrophobic performance. During the filtration process, wet particles accumulate on the surface of the nanofibrous membrane and subsequently break off. The membrane’s porosity and pressure drop remain unchanged. However, adding fluorinated polyimide significantly improves the filtration performance of the nanofibrous membrane while maintaining a filtration efficiency of 99.0%. This improvement is achieved by increasing the proportion of F-PI.

**Figure 7:**
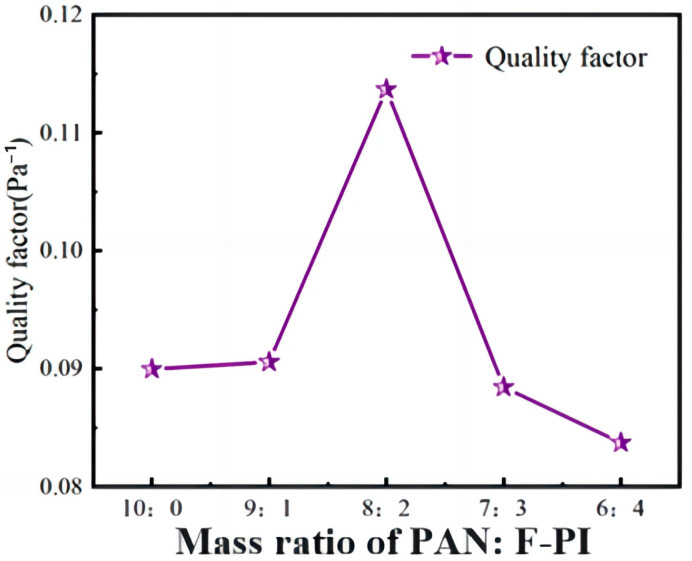
Quality factor of nanofibrous membranes prepared with different PAN:F-PI ratios.

**Figure 8:**
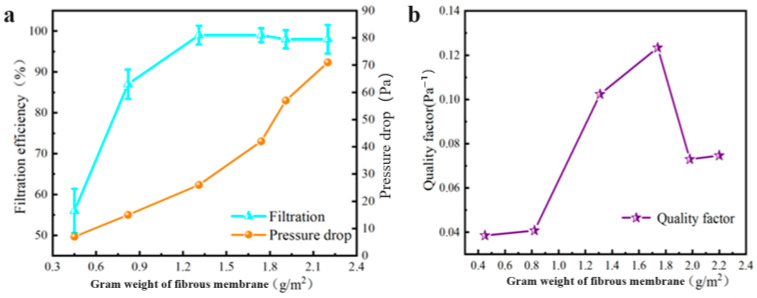
(**a**) Filtration performance and (**b**) quality factor of nanofibrous membranes prepared with different gram weights.

The addition of F-PI significantly improved the filtration performance of the nanofibrous membrane, although it did result in a slight increase in resistance pressure drop. The filtration resistance pressure drop of the pure PAN was 18 Pa. When the PAN and F-PI mass ratio varied (9:1, 8:2, 7:3, 6:4), the resistance pressure drop increased to 36 Pa, 45 Pa, 47 Pa, and 51 Pa, respectively. This increase in resistance pressure drop can be attributed to the change in nanofibrous membrane diameter. As more F-PI is added, the resulting finer fiber diameter leads to lower porosity and a slight increase in pressure drop. The quality factor is a crucial parameter for evaluating the filtration performance of a nanofibrous membrane. Figure 7 shows the calculated quality factor of the nanofibrous membrane. It can be observed that the maximum quality factor of 0.114 pa^−1^ is achieved when the addition amount is 8:2. The correlation properties of the nanofibrous membrane with different base weights were explored based on this mass ratio. Nanofibrous membranes with weights of 0.45, 0.82, 1.31, 1.74, 1.91, and 2.2 g·m^−2^ were prepared and subjected to relevant filter tests, as shown in Figure 8.

Figure 8 shows that the filtration performance of the nanofibrous membrane improves as the gram weight increases, but the resistance pressure drop also increases linearly. The F-PI/PAN composite nanofibrous membrane can remove 50.3 ± 5.4% of PM_2.5_ when the gram weight is 0.5 g/m^2^, and the filtration efficiency can reach 76.0 ± 2.1% when the gram weight is 0.82 g/m^2^. When the weight of the PI/PAN composite nanofibrous membrane exceeds 1 g/m^2^, the filtration efficiency is significantly improved. The highest quality factor was achieved when the fiber film weight was 1.74 g·m^−2^, which was 0.123 pa^−1^.

**Figure 9:**
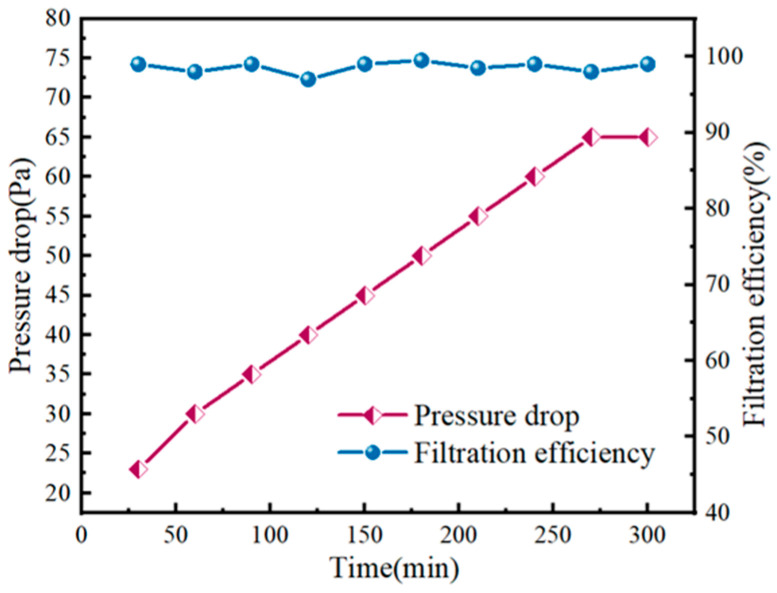
Recycling performance of nanofibrous membrane prepared with PAN:F-PI = 8:2 and 1.74 g/m^2^ g weight.

To investigate the performance of the nanofibrous membrane, the change in resistance pressure drop during filtration was studied. Figure 9 shows the change in resistance pressure drop of the nanofibrous membrane within 300 min. The pressure drop gradually increases with the extension of filtration time, and the resistance pressure drop increases linearly between 0 and 250 min. At 250 min, the resistance pressure drop is 65 Pa, and then it remains stable for 60 min. It takes 300 min to reach the maximum dust capacity at ultra-high concentrations.

### 3.5. Mechanical Properties of Fluorinated PI/PAN Nanofibrous Membranes

**Figure 10:**
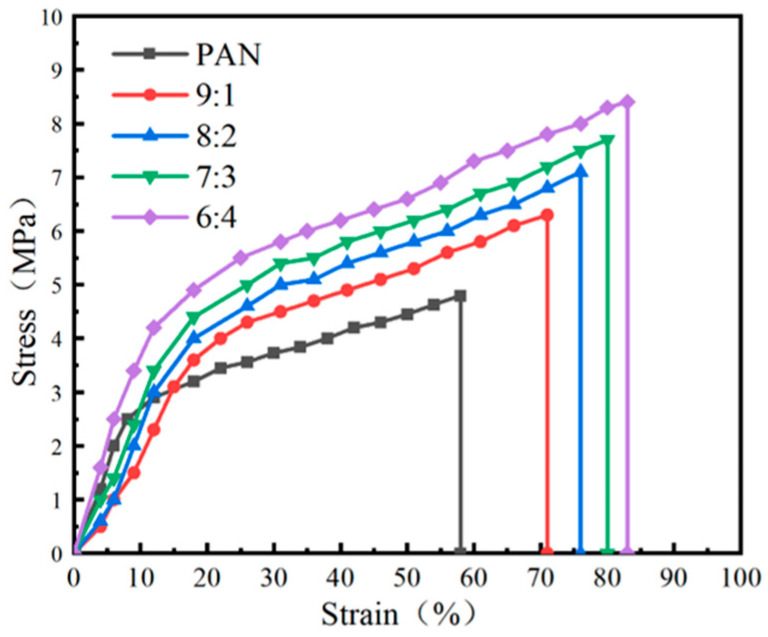
The mechanical properties of nanofibrous membranes prepared with different PAN:F-PI ratios.

The mechanical properties of fluorinated PI/PAN nanofibrous membranes were tested using a universal tensile testing machine. Figure 10 presents the results indicating that adding F-PI improves the nanofibrous membranes’ mechanical properties. The inclusion of F-PI, which contains numerous nitrogen-containing five-membered heterocyclic and aromatic rings, led to the creation of inflexible molecular chains, thereby enhancing the mechanical strength and elongation at the break of the nanofibrous membranes. At a mass ratio of 9:1, the elongation at break is 71%, and the breaking strength is 6.3 MPa. At a mass ratio of 6:4, the elongation at break is 83%, and the breaking strength is 8.4 MPa. In addition, the fracture strength of the nanofibrous membranes was improved, and their elongation at break was increased. This was due to the increased number of solution-binding sites after mixing.

## 4. Conclusions

In this paper, F-PI/PAN nanofibrous membranes were successfully prepared by electrospinning. The nanofibrous membranes exhibited good thermal stability up to 300 °C. The contact angle of the nanofibrous membrane increased with the addition of F-PI content, reaching a maximum of 128 ± 5.2° at an addition ratio of 6:4. Furthermore, the PM_2.5_ filtration efficiency of the nanofibrous membrane was tested. The study discovered that adding F-PI significantly improved the filtration efficiency of the nanofibrous membrane. At a mass ratio of 8:2, the membrane achieved a filtration efficiency of 99.4 ± 0.3%. Furthermore, the mechanical properties of the composite nanofibrous membrane improved progressively with an increasing F-PI mass ratio. The fracture strength increased, and the elongation at break also increased accordingly. When the mass ratio is 8:2, the nanofibrous membrane elongates at a break of 76% and a fracture strength of 7.1 MPa. This is 1.63 times greater than that of the pure PAN membrane. Based on a comprehensive analysis of the characterization results, PAN:F-PI = 8:2 is the best ratio for preparing nanofiber air filtration membranes. At this ratio, the quality factor of the fiber membrane is as high as 0.114 Pa^−1^, showing the best filtration performance and mechanical strength. This method is feasible for improving air filtration efficiency and has great potential for application.

## Data Availability

Data are contained within the article.

## References

[1] Wu Y., Wang W., Liu C., Chen R., Kan H. (2020). The association between long-term fine particulate air pollution and life expectancy in China, 2013 to 2017. Sci. Total Environ..

[2] Chan C.K., Yao X. (2008). Air pollution in mega cities in China. Atmos. Environ..

[3] Liu J., Wu T., Liu Q., Wu S., Chen J.C. (2020). Air pollution exposure and adverse sleep health across the life course: A systematic review. Environ. Pollut..

[4] Chen Y., Shen H., Smith K.R., Guan D., Chen Y., Shen G., Liu J., Cheng H., Zeng E.Y., Tao S. (2018). Estimating household air pollution exposures and health impacts from space heating in rural China. Environ. Int..

[5] Liu C., Hsu P.C., Lee H.W., Ye M., Zheng G., Liu N., Li W., Cui Y. (2015). Transparent air filter for high-efficiency PM2.5 capture. Nat. Commun..

[6] Dai X., Liu J., Li X., Zhao L. (2018). Long-term monitoring of indoor CO_2_ and PM2.5 in Chinese homes: Concentrations and their relationships with outdoor environments. Build. Environ..

[7] Maher B.A., O’Sullivan V., Feeney J., Gonet T., Kenny R.A. (2021). Indoor particulate air pollution from open fires and the cognitive function of older people. Environ. Res..

[8] Yüksekkaya M.E., Tercan M., Doğan G. (2010). Filter media research: Fabric reinforcement of nonwoven filter cloths. Filtr. Sep..

[9] Park J.H., Yoon K.Y., Na H., Kim Y.S., Hwang J., Kim J., Yoon Y.H. (2011). Fabrication of a multi-walled carbon nanotube-deposited glass fiber air filter for the enhancement of nano and submicron aerosol particle filtration and additional antibacterial efficacy. Sci. Total Environ..

[10] Park H.S., Park Y.O. (2005). Filtration properties of electrospun ultrafine fiber webs. Korean J. Chem. Eng..

[11] Su Y., Zhang Z., Wang Z., Chen M., Dong M., Han X. (2017). Necklace-like fiber composite membrane for high-efficiency particulate matter capture. Appl. Surf. Sci..

[12] Amin A., Merati A.A., Bahrami S.H., Bagherzadeh R. (2017). Effects of porosity gradient of multilayered electrospun nanofibre mats on air filtration efficiency. J. Text. Inst..

[13] Wu W., Sota H., Hirogaki T., Aoyama E. (2021). Investigation of air filter properties of nanofiber non-woven fabric manufactured by a modified melt-blowing method along with flash spinning method. Precis. Eng..

[14] Greiner A., Wendorff J.H. (2007). Electrospinning: A fascinating method for the preparation of ultrathin fibers. Angew. Chem. Int. Ed..

[15] Meng H., Xu T., Gao M., Bai J., Li C. (2021). An oil-contamination-resistant PVP/PAN electrospinning membrane for high-efficient oil–water mixture and emulsion separation. J. Appl. Polym. Sci..

[16] Shou D.H., He J.H. (2008). PAN/PVP micro composite fibers using electrospinning. J. Polym. Eng..

[17] Linyu M.E.I., Xigang W., Yaqing L.I.U., Junyuan W.A. (2019). Computer simulation of PAN/PVP blends compatibility and preparation of aligned PAN porous nanofibers via magnetic-field-assisted electrospinning PAN/PVP blends. Mater. Sci..

[18] Shang L., Li L., Li Y., Yang C. (2016). Preparation of composite polyimide filter felt and its removal of PM2.5 particles. Chin. J. Process Eng..

[19] Gao X., Li Z.K., Xue J., Qian Y., Zhang L.Z., Caro J., Wang H. (2019). Titanium carbide Ti_3_C_2_T_x_ (MXene) enhanced PAN nanofiber membrane for air purification. J. Membr. Sci..

[20] Raju S., Siddharthan T., McCormack M.C. (2020). Indoor air pollution and respiratory health. Clin. Chest Med..

[21] Ding J., Zhang G., Dai H., Chen H., Fu H. (2024). Gas Sensor Preparation Based on Green Biological Template: A Review. Sens. Actuators A Phys..

[22] Xiao Y., Lyu H., Yang C., Zhao B., Wang L., Tang J. (2021). Graphitic carbon nitride/biochar composite synthesized by a facile ball-milling method for the adsorption and photocatalytic degradation of enrofloxacin. J. Environ. Sci..

[23] Faisal M., Jalalah M., Harraz F.A., El-Toni A.M., Khan A., Al-Assiri M.S. (2020). Au nanoparticles-doped g-C_3_N_4_ nanocomposites for enhanced photocatalytic performance under visible light illumination. Ceram. Int..

[24] Ma Q., Kutilike B., Kari N., Abliz S., Yimit A. (2021). Study on surface sensitization of g-C_3_N_4_ by functioned different aggregation behavior porphyrin and its optical properties. Mater. Sci. Semicond. Process..

[25] Liu J., Zhang H., Gong H., Zhang X., Wang Y., Jin X. (2019). Polyethylene/polypropylene bicomponent spunbond air filtration materials containing magnesium stearate for efficient fine particle capture. ACS Appl. Mater. Interfaces.

[26] Li C., Wang Y., Li C., Xu S., Hou X., Wu P. (2019). Simultaneously broadened visible light absorption and boosted intersystem crossing in platinum-doped graphite carbon nitride for enhanced photosensitization. ACS Appl. Mater. Interfaces.

[27] Yakovleva O.I., Sashina E.S., Nabieva I.A. (2020). Modification of polyacrylonitrile fibers with fibroin nanoparticles. Production and attachment of fibroin nanoparticles to PAN fiber. Fibre Chem..

[28] Fu S., Yu Q., Liu Z., Hu P., Chen Q., Feng S., Mai L., Zhou L. (2019). Yolk–shell Nb_2_O_5_ microspheres as intercalation pseudocapacitive anode materials for high-energy Li-ion capacitors. J. Mater. Chem. A.

[29] Wang Z., Chen L., Wang Y., Chen X., Zhang P. (2016). Improved cell adhesion and osteogenesis of op-HA/PLGA composite by poly (dopamine)-assisted immobilization of collagen mimetic peptide and osteogenic growth peptide. ACS Appl. Mater. Interfaces.

[30] Li L., Zhang J., Li Y., Yang C. (2017). Removal of Cr (VI) with a spiral wound chitosan nanofiber membrane module via dead-end filtration. J. Membr. Sci..

[31] Biffinger J.C., Kim H.W., DiMagno S.G. (2004). The polar hydrophobicity of fluorinated compounds. ChemBioChem.

[32] Dalvi V.H., Rossky P.J. (2010). Molecular origins of fluorocarbon hydrophobicity. Proc. Natl. Acad. Sci. USA.

[33] Li S., Jinjin D. (2007). Improvement of hydrophobic properties of silk and cotton by hexafluoropropene plasma treatment. Appl. Surf. Sci..

[34] Liang L., Lis Arias M.J., Lou Z., Mao Q., Ye C., Meng X. (2019). Preparation of hydrophobic fabrics and effect of fluorine monomers on surface properties. J. Eng. Fibers Fabr..

[35] Park S.H., Kim J.H., Moon S.J., Drioli E., Lee Y.M. (2018). Enhanced, hydrophobic, fluorine-containing, thermally rearranged (TR) nanofiber membranes for desalination via membrane distillation. J. Membr. Sci..

[36] Myeong S., Lim C., Kim S., Lee Y.S. (2023). High-efficiency oil/water separation of hydrophobic stainless steel Mesh filter through carbon and fluorine surface treatment. Korean J. Chem. Eng..

